# Bioresorbable Materials for Wound Management

**DOI:** 10.3390/biomimetics10020108

**Published:** 2025-02-12

**Authors:** Hye-Min Lee, Hanjun Ryu

**Affiliations:** 1 Department of Advanced Materials Engineering, Chung-Ang University, Anseong-si 17546, Gyeonggi-do, Republic of Korea; 2 Department of Intelligence Energy and Industry, Chung-Ang University, Seoul 06974, Republic of Korea

**Keywords:** bioresorbable materials, electrical stimulation, drug delivery, wound healing, diabetic

## Abstract

Chronic wounds pose a significant healthcare challenge due to their risk of severe complications, necessitating effective management strategies. Bioresorbable materials have emerged as an innovative solution, offering advantages such as eliminating the need for secondary surgical removal, reducing infection risks, and enabling time-delayed drug delivery. This review examines recent advancements in bioresorbable wound healing materials, focusing on a systematic review of bioresorbable materials, systems incorporating electrical stimulation, and drug delivery technologies to accelerate tissue repair. The discussion encompasses the fundamental principles of bioresorbable materials, including their resorption mechanisms and key properties, alongside preclinical applications that demonstrate their practical potential. Critical challenges impeding widespread adoption are addressed, and prospects for integrating these cutting-edge systems into clinical practice are outlined. Together, these insights underscore the promise of bioresorbable materials in revolutionizing chronic wound care.

## 1. Introduction

Chronic wounds, often resulting from metabolic disorders such as diabetic foot ulcers and venous ulcers, present significant health and economic challenges in modern society [1]. These wounds lead to severe complications, including persistent inflammation, pain, and reduced mobility, with some cases progressing to amputation [2]. Chronic wounds affect 1–2% of individuals in developed countries, a figure expected to rise sharply due to the aging global population [3]. By 2025, the diabetic population worldwide is projected to exceed 400 million, further exacerbating the burden of prolonged wound healing [4]. Economically, chronic wounds impact over 6.5 million patients annually in the United States alone, contributing to healthcare costs estimated at $25 billion [5]. These figures underscore the urgent need for innovative therapies beyond conventional treatments like bandages and dressings [6].

Innovative strategies such as regulatory drug release, tissue regeneration technology, and advances in regenerative medicine have shown significant potential in promoting continuous wound recovery [7,8]. Among these, electric fields have been identified as an effective method to promote wound repair by enhancing epithelial cell migration and overall healing processes [9]. Studies indicate that electric stimulation not only accelerates cellular migration but also guides its directionality [10]. Pulsed electric field stimulation has been shown to foster the proliferation of fibroblasts and endothelial cells, expediting tissue regeneration [11]. Additionally, electric stimulation increases the mobility of macrophages and neutrophils, activates fibroblasts, and supports the wound-healing cascade [12]. Complementing these advancements, intelligent responsive drug delivery systems provide controlled and efficient drug administration [13,14]. These platforms are characterized by critical attributes such as optimal mechanical strength, self-repair functionality, and strong adhesion to tissues. They facilitate the controlled delivery of therapeutic compounds, providing antioxidant, antimicrobial, anti-inflammatory, and angiogenic benefits. Additionally, they modulate macrophage polarization to enhance the wound healing process [15]. Bioresorbable materials play a pivotal role in these advanced therapies. Their unique properties, including bioresorption within the body, environmental friendliness, and the elimination of surgical removal procedures, make them highly advantageous [16,17]. Moreover, byproducts formed during the bioresorbing process act as physical barriers and create a microenvironment conducive to wound healing, garnering significant attention in the medical field [18].

The field of biomimetics has also driven innovation in bioresorbable electronics and devices for wound healing [19]. For example, bioresorbable polymers and metals are inspired by biological systems that maintain structural integrity while adapting to their environment [20]. Similarly, triboelectric nanogenerators (TENGs) mimic the energy conversion processes found in biological systems, such as the mechanotransduction seen in cell membranes [21]. By integrating these biomimetic principles, this review explores how bioresorbable materials, combined with TENG-based systems, offer transformative potential in wound healing through electrical stimulation and drug delivery. These developments are poised to bridge the gap between biology and engineering, advancing personalized and efficient therapeutic solutions.

**Figure 1:**
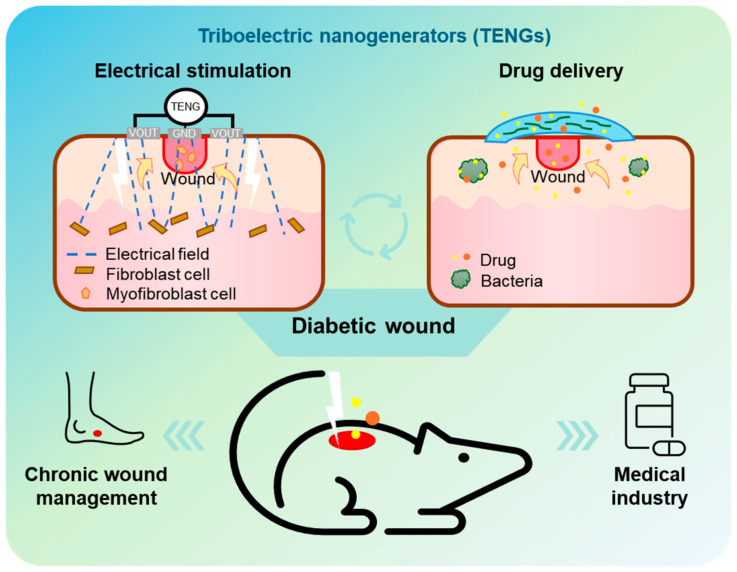
Overview of this review. Fundamental mechanism of bioresorption and applications in wound healing (electrical stimulation and drug delivery).

This review summarizes the fundamental mechanisms of bioresorption in materials such as metals, polymers, and semiconductors, with a focus on their key properties, including electrical characteristics and resorption rates. Based on these foundations, recent applications of bioresorbable materials in wound healing are analyzed, particularly in the context of electrical stimulation and drug delivery systems. In particular, bioresorbable materials-based TENGs for wound healing applications are comprehensively reviewed as emerging electroceuticals (Figure 1) [22,23,24,25]. Finally, prospects for these technologies are discussed, summarizing recent research trends and highlighting the potential to revolutionize chronic wound management and become integral to the medical industry.

## 2. Bioresorbable Materials

Bioresorbable materials can be broadly classified into three main types: metals, polymers, and semiconductors [26]. Each category exhibits its own chemical and physical properties, making them suitable for chronic wound treatment using electrical stimulation and drug delivery effects. For instance, metals like magnesium (Mg) are often used for their excellent conductivity, polymers for their flexibility and tunable bioresorption rates, and semiconductors for enabling electronic functionalities in bioresorbable devices.

### 2.1. Metal

#### 2.1.1. Bioresorbable Mechanism of Metals

**Figure 2:**
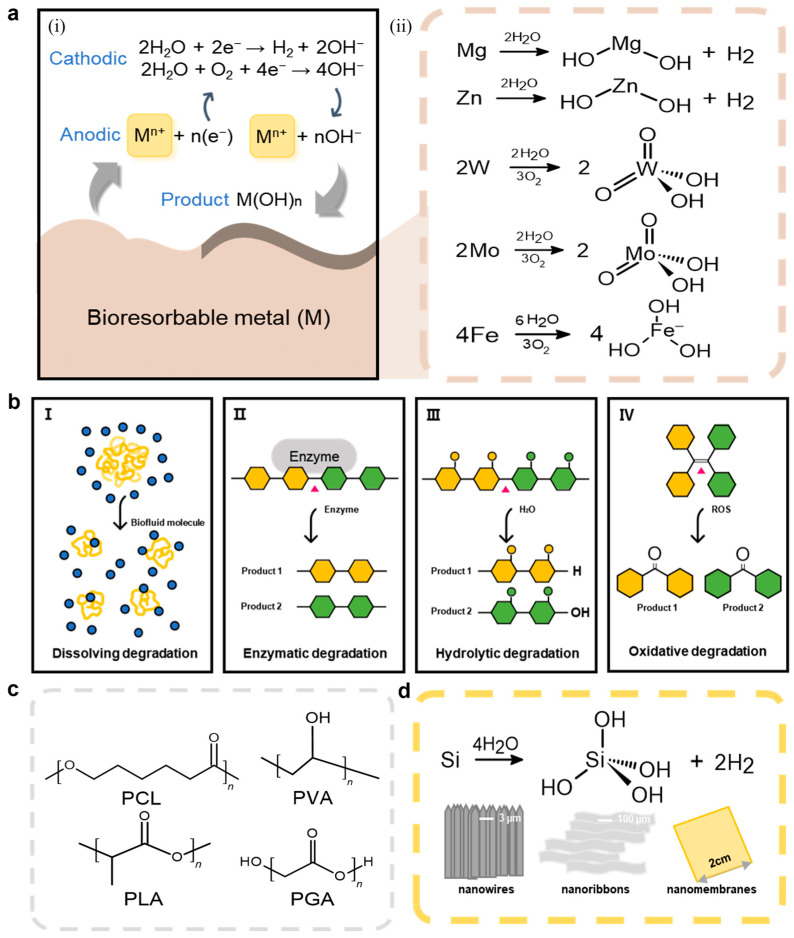
Bioresorbable materials. (**a**) Schematic illustration of the chemical reactions involved in the dissolution of bioresorbable metals (M: Mg, Zn, W, MO, Fe). (**b**) Schematic illustrations of the representative mechanisms involved in the in vivo resorption of bioresorbable polymers (PCL, PVA, PLA, PGA). (**c**) PCL, PVA, PLA, and PGA chemical structure in 2D. (**d**) Schematic illustration of the bioresorption process of silicon nanostructures (silicon nanowires, nanoribbons, nanomembranes) in bodily fluids.

Bioresorbable metals, known for their excellent conductivity, are integral to various electronic medical devices [27]. Examples of these metals include bioresorbable inorganic conductors, such as alkali-earth metals like Mg [28], transition metals including molybdenum (Mo) [29], tungsten (W) [30], zinc (Zn) [31], and iron (Fe) [32], as well as their alloys, such as the Mg-based alloy AZ31B [33]. These materials are employed in diverse forms, including micrometer-scale thin films, foils, particles, wires, or composites integrated with bioresorbable polymers [34]. The bioresorption of these metals is governed by electrochemical processes involving anodic and cathodic reactions (Figure 2a(i)) [26]. When exposed to water or biological fluids, electrochemical oxidation occurs, producing metal cations (M^n+^) and electrons (e^−^) via the anodic reaction [35]

M → M^n+^ + ne^−^

Simultaneously, byproducts such as hydroxide ions (OH^−^), hydrogen gas (H_2_), metal hydroxides (M(OH)_n_), and phosphates are generated during cathodic reactions. For example, in the anodic processes involving Mg and Zn, the generated electrons contribute to water reduction [36]:

2H_2_O + 2e^−^ → H_2_ + 2OH^−^

This reaction leads to the formation of hydrogen gas and metal hydroxides, which create surface layers on the metal. However, these protective layers are progressively resorbed by chloride ions, reactive species, or biochemical components in body fluids, such as proteins, lipids, and amino acids, leading to ongoing metal dissolution. In contrast, metals such as Fe, Mo, and W undergo oxygen reduction instead of hydrogen gas generation [37]:

2H_2_O + O_2_ + 4e^−^ → 4OH^−^

This reaction forms dense surface layers, reducing the resorption rates compared to Mg and Zn. The following chemical reactions summarize the specific bioresorption pathways for each metal (Figure 2a(ii)) [38]:

Mg + 2H_2_O → Mg(OH)_2_ + H_2_

Zn + 2H_2_O → Zn(OH)_2_ + H_2_

2W + 2H_2_O + 3O_2_ → 2H_2_WO_4_

2Mo + 2H_2_O + 3O_2_ → 2H_2_MoO_4_

4Fe + 3O_2_ + 6H_2_O → 4Fe(OH)_3_

#### 2.1.2. Properties of Metals

**Figure 3:**
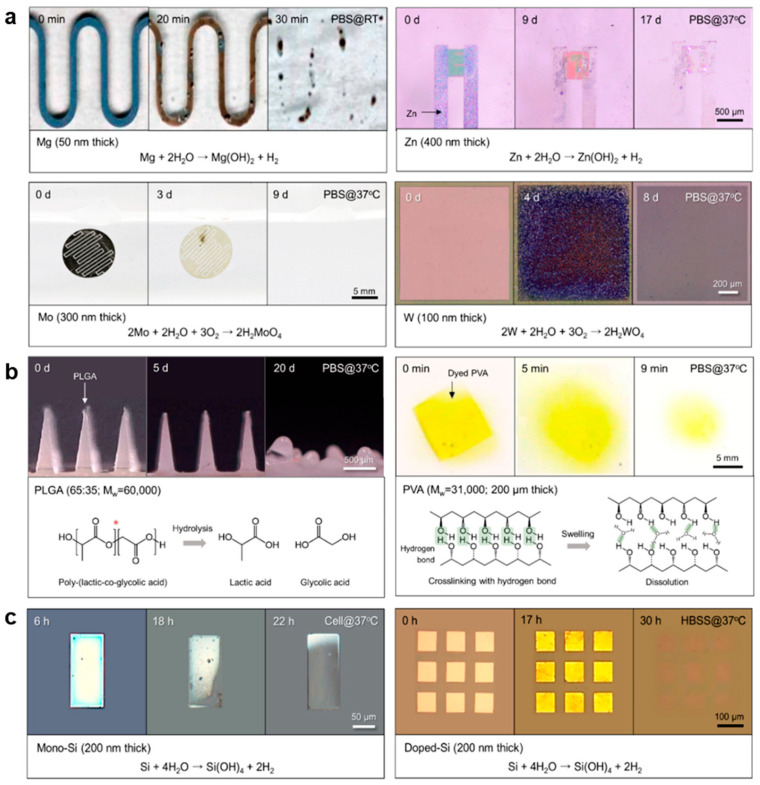
Chemical reactions involved in the dissolution of bioresorbable metals, polymers, and semiconductors [26]. (**a**) Series of images illustrating the dissolution of metal material patterns (**top**) and the corresponding reactions (**bottom**). (**b**) Series of images showing the dissolution of polymer material patterns (**top**) and the related reactions (**bottom**). (**c**) Series of images depicting the dissolution of semiconductor material patterns (**top**) and the associated reactions (**bottom**). Reprinted with permission from Zhang et al.; Copyright © 2023 American Chemical Society.

Bioresorbable metals exhibit excellent electrical conductivity and adjustable resorption rates, making them ideal for use as electrodes and interconnects in electronic systems [39]. For instance, metals such as Mg, Zn, Mo, W, and Fe exhibit excellent conductivity, with values at 20 °C as follows: Mg (2.3 × 10^7^ S m^−1^), Zn (1.7 × 10^7^ S m^−1^), Mo (2.0 × 10^7^ S m^−1^), W (2.0 × 10^7^ S m^−1^), and Fe (1.0 × 10^7^ S m^−1^) (Figure 3a) [26,40].

The resorption rates of these metals have been evaluated in Hank’s Balanced Salt Solution (HBSS) at pH 7.4 and 37 °C. Thin films (50 nm to 400 nm in thickness) of Mg, Zn, Mo, W, and Fe were tested, showing the following resorption rates: 480, 300, 20, 0.7, and 7 nm/day, respectively [41]. Similarly, when tested in phosphate-buffered saline (PBS) under the same pH and temperature conditions, the resorption rates for 50 µm-thick foils of Mg, Zn, W, Mo, and Fe were 4000, 3500, 150, 20, and 5–80 nm/day, respectively [42]. Further analysis was conducted on bioresorbable thin films made from Mg, AZ31B (a Mg alloy), Zn, Fe, Mo, and W, each with a serpentine pattern (250 µm wide, 50 mm long). The thicknesses of these films varied, ranging from 40 nm (Mo) to 300 nm (Mg, AZ31B, Zn, Fe) and 150 nm (W). The results showed that Mg, AZ31B, and Zn films exhibited uneven corrosion, leading to localized defects and electrical disconnection [43]. These metals had electrical dissolution rates (EDRs) more than 10 times higher than their corrosion rates (CR). In contrast, Mo and W films displayed uniform surface corrosion, and due to the slower resorption of their oxide layers (MoO_x_, WO_x_), the EDRs were 2–3 times the CR [44].

### 2.2. Polymer

#### 2.2.1. Bioresorbable Mechanism of Polymers

Conductive polymers (CPs) present a compelling alternative to conventional metals, owing to their superior processability and desirable mechanical characteristics [45]. These materials are generally synthesized by doping conjugated polymers, where π-electrons are delocalized along their molecular backbone (Figure 2b,c) [26]. Although the majority of CPs maintain chemical stability in physiological environments and are typically non-bioresorbable, advanced design and fabrication techniques can achieve varying levels of bioresorbability [46].

One approach involves blending CPs with bioresorbable non-CPs, resulting in partially bioresorbable conductors [47]. This strategy is similar to the use of infiltrative composites with metals [48]. Examples include blending polypyrrole (PPy), polyaniline (PANI), or poly(3,4-ethylenedioxythiophene) (PEDOT) with bioresorbable polymers such as poly(lactic-co-glycolic acid) (PLGA), poly(D,L-lactic acid) (PDLLA), poly(L-lactic acid) (PLLA), polycaprolactone (PCL), or silk fibroin (SF) [49]. A second method entails introducing ionizable functional groups into CPs or blending modified CPs with bioresorbable polymers to create completely bioresorbable conductive polymers [50]. In the third approach, electroactive oligomer units, such as pyrrole-thiophene-pyrrole oligomers, aniline oligomers, or aniline trimer, tetramer, and pentamer, are integrated with bioresorbable polymers like PLA, PCL, PLLA, or PU [51]. These are further enhanced by incorporating bioresorbable functional groups, such as hydrolyzable ester bonds [52].

Non-CPs used as substrates—such as PCL, polyvinyl alcohol (PVA), polylactic acid (PLA), and polyglycolic acid (PGA)—resorb through four primary mechanisms of (1) dissolving degradation (where solvated polymer chains interact with water molecules), (2) enzymatic degradation (enzymes catalyze the breakdown of polymer chains), (3) hydrolytic degradation (water molecules induce depolymerization through chemical reactions), and (4) oxidative degradation (reactive oxygen or nitrogen species (ROS/RNS) drive depolymerization) to achieve bioresorbability [53]. Non-CPs primarily resorb via hydrolysis, producing byproducts such as lactic acid, glycolic acid, and 6-hydroxyhexanoic acid. These byproducts enter the tricarboxylic acid (TCA) cycle and are eventually metabolized into carbon dioxide (CO_2_) and water (H_2_O).

#### 2.2.2. Properties of Polymers

CPs generally exhibit higher electrical resistance than metals, but their mechanical properties, such as elongation, make them more suitable for applications in biological materials (Figure 3b) (Table 1) [26,54]. One approach to enhancing the conductivity of CPs involves doping conjugated polymers with conductive substances. For example, FeCl_3_-doped PPy nanoparticles were incorporated into PDLLA at 3 wt%, resulting in an electrical conductivity of 1.0 × 10^−3^ S m^−1^ [55,56]. Another method, which involves electrochemically coating PPy onto PLGA or PLLA fibers, increased the electrical conductivity from 1.0 × 10^−16^ S m^−1^ to 1.0 × 10^−4^ S m^−1^ [57,58]. Under physiological conditions (PBS, pH 7.4, 37 °C), these fibers experienced a 14–24% weight loss after 12 weeks. Similarly, when PPy was directly coated onto an SF substrate, both the silk and PPy underwent resorption, with an 82% weight loss after 15 days under the same conditions [59]. Another example involves PANI doped with camphor sulfonic acid and electrospun with gelatin or poly(L-lactide-co-ε-caprolactone), forming fiber sheets [60]. At 30 wt%, these fibers exhibited electrical conductivities ranging from 1.4 to 2.1 × 10^−2^ S m^−1^. Additionally, PEDOT, doped with hyaluronic acid (PEDOT-HA) at 10 wt%, and loaded onto PLLA, demonstrated an electrical conductivity of 4.7 × 10^−3^ S m^−1^. Due to the water affinity of the HA domain, water penetration increased, leading to a resorption rate of 10% after 8 weeks [61]. A second method involves modifying CPs with ionizable or hydrolyzable groups for resorption. For example, PPy-based films (500 µm thick) polymerized via electrochemical oxidation and ferric chloride (III) showed 100% mass loss within 24 h at pH 8.2 and 37 °C, and 27% mass loss after 80 days at pH 7.2, 37 °C [62]. Additionally, multilayer films assembled using layer-by-layer technology, composed of poly(ammonium(3-thienyl)ethoxypropanesulfonate) (SPT) and poly(ethylenimine) (PEI), exhibited an electrical conductivity of 2.76 × 10^−2^ S m^−1^ and fully resorbed at pH 7.4, 37 °C [63]. A third method involves creating bioresorbable conductive materials, such as bioresorbable conductive polyurethane (BCPU), which is a copolymer of aniline trimer (electroactive unit) and PCL (bioresorbable unit). BCPU demonstrated an electrical conductivity of 1.2–5.5 × 10^−5^ S m^−1^ and experienced 12–14% mass loss after 8 weeks in PBS at 37 °C due to hydrolytic resorption [64]. Similarly, poly[(glycine ethyl ester)(aniline pentamer) phosphazene] (PGAP), composed of aniline pentamer (electroactive unit), glycine ethyl ester (bioresorbable unit), and polyphosphazene (chemical linker), exhibited an electrical conductivity of 2 × 10^−5^ S m^−1^ and underwent 50% mass loss after 70 days in vitro (PBS, pH 7.4, 37 °C) due to the hydrolysis of ester bonds [65].

The resorption rates of non-conductive polymers, such as PCL [66], PVA [67], PGA [68], and PLA [69], under physiological conditions (PBS, pH 7.4, 37 °C) are as follows: 3% after 5 weeks for PCL, 100% after 30 min for PVA, 100% after 3 weeks for PGA, and 64% after 12 months for PLA. The resorption rate is controlled by the composition of the copolymer. For instance, in PLGA, adjusting the ratio of lactic acid to glycolic acid (75:25, 65:35, 50:50) results in complete weight loss within 50, 40, and 30 days, respectively [70].

### 2.3. Semiconductor

#### 2.3.1. Bioresorbable Mechanism of Semiconductors

While bulk silicon wafers are typically regarded as non-resorbable due to their significant thickness (usually in the millimeter range) and the natural oxide layer on their surface, silicon nanostructures such as nanowires, nanoribbons, and nanomembranes are capable of completely dissolving in bodily fluids over time [71]. The dissolution rate of these nanostructures is influenced by their geometry and surface chemical properties [72]. Bioresorption of silicon occurs through a chemical reaction with surrounding water, resulting in the production of orthosilicic acid (Si(OH)_4_) and hydrogen gas (H_2_) (Figure 2d) [26]. The chemical reaction is represented as follows [73]:

Si + 4H_2_O → Si(OH)_4_ + 2H_2_

#### 2.3.2. Properties of Semiconductors

The use of silicon-on-insulator silicon wafers facilitates the fabrication of ultra-thin silicon films, enabling the development of electronic devices for diverse applications (Figure 3c) [26,74]. In particular, single-crystal silicon (mono-Si) films with a thickness of 100 nm exhibit a dissolution rate of 2–5 nm/day in PBS (0.1 M, pH 7.4, 37 °C) [75]. A 200 nm-thick mono-Si film is fully resorbed under body temperature and cell culture conditions without negatively impacting cellular metabolism [76]. The hydrolysis behavior of silicon nanostructures depends significantly on doping type and concentration [77]. For instance, when a 70 nm-thick silicon nanomembrane was immersed in PBS (0.1 M, pH 7.4, 37 °C), n-type silicon dissolved at a rate of 3.1 nm/day, while p-type silicon exhibited a slightly slower rate of 2.9 nm/day at doping concentrations of 10^17^ to 10^19^ cm^−3^ [78]. However, as the doping concentration increased to ~10^20^ cm^−3^ or higher, the dissolution rate markedly decreased to 0.4 nm/day for n-type and 0.2 nm/day for p-type silicon, indicating an inverse relationship between doping concentration and dissolution rate [79]. The dissolution rate of silicon nanostructures is also highly sensitive to the chemical composition of the surrounding solution, with factors such as solution type, added compounds, and pH levels playing critical roles [80]. For example, when tested in HBSS (pH 7.6, 37 °C) and bovine serum (pH 7.4, 37 °C), the dissolution rates were 58 nm/day and 21 nm/day, respectively, underscoring the substantial influence of the solution type on the dissolution behavior [81].

## 3. Applications of Bioresorbable Materials in Wound Management

Bioresorbable materials have enabled the development of innovative medical devices across various applications, including biological activity sensors, electrical stimulation therapies, and implantable devices [82]. Their inherent bioresorption properties—allowing materials to resorb and be absorbed by the body—eliminate the need for device retrieval after therapy, providing significant clinical benefits [83]. For implantable devices, particularly those not mounted on the skin, bioresorbable electrodes used for direct electrical stimulation can be placed within wounds, beneath the regenerated skin layer [84]. This approach not only enhances therapeutic outcomes but also prevents additional tissue damage that might occur during device removal. Additionally, bioresorbable materials serve as versatile platforms for long-term, controlled drug delivery. They enable passive drug release over extended periods while also supporting active release mechanisms triggered by external stimuli, such as heat, changes in pH, or electrical stimulation [85]. These functionalities expand the potential of bioresorbable materials to address complex wound management needs effectively.

### 3.1. Based on Electric Stimulation Effects

#### 3.1.1. Mo-Based Bioresorbable, Wireless, Battery-Free Electrical Therapy Systems (BESs)

**Figure 4:**
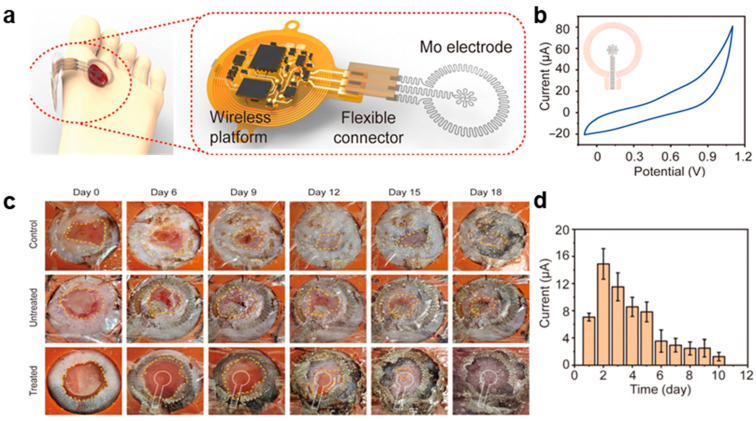
Mo-based bioresorbable, wireless, battery-free electrical therapy systems (BESs) [86]. (**a**) Schematic illustration of Mo-based bioresorbable, wireless, battery-free electrical therapy systems (BESs). (**b**) CV of the Mo electrode in DPBS solution. (**c**) In vivo experimental results. (**d**) In vivo wireless current monitoring results between Mo electrodes. Reprinted with permission from Song et al.; Copyright © 2023 The American Association for the Advancement of Science.

Mo-based bioresorbable, wireless, battery-free electrical therapy systems (BESs) represent an innovative approach to wound treatment, providing electrical stimulation and impedance measurement (Figure 4a) [86]. The BES consists of key components: a wireless platform, a flexible connector, and a pair of Mo electrodes. Serpentine design of Mo electrodes allow for elastic behavior up to a 9% stretch, ensuring skin-like stretchability with interfacial stress below sensory thresholds for stability [87]. While plastic deformation occurs at 20–30% stretch, the strain remains minimal (0.2%) under bending and torsion, demonstrating excellent flexibility. The electric field strength near the inner Mo electrode is ~250 mV/mm, a condition proven to accelerate wound healing [88,89]. As the wound heals, the inner electrodes embed within the regenerated skin and subsequently undergo bioresorption, eliminating the need for surgical removal. The dissolution process follows the following reactions [90]:

Mo + 2H_2_O + O_2_ → MoO_4_^2−^ + 4H^+^

MoO_3_ + H_2_O → MoO_4_^2−^ + H^+^

In accelerated bioresorption tests (75 °C in DPBS, pH 7.4), which simulate a degradation rate ~16 times faster than body temperature, Mo foil begins deforming at 12 days and nearly dissolves by 18 days. At 37 °C, dissolution rates measured by ICP-MS and weight analysis range from 50 to 70 nm/day, consistent with physiological conditions. The electrical performance and stability of the Mo electrodes were validated through cyclic voltammetry (CV) analysis, which confirmed stable redox reactions within a voltage range of 0–1.1 V (Figure 4b) [86].

It was hypothesized that Mo electrodes would accelerate keratinocyte migration in vitro and generate an endogenous electric field within the wound center, thereby enhancing the wound healing response in vivo. Results revealed that electrical stimulation reduced suturing time by ~30% and significantly enhanced wound recovery (Figure 4c) [86]. In the treated group, wound size reduced to 86.0 ± 10% within 15 days, with complete healing achieved in just over three weeks. In contrast, the untreated and control groups required over four weeks to heal. Initially, the wound exhibited an electrically conductive environment (~20 μA), but as the wound dried and ion conductivity decreased, the current dropped to 0 μA (Figure 4d) [86]. The bioresorption and biocompatibility of Mo electrodes were further assessed through micro-computed tomography (micro-CT). Internal Mo electrodes persisted for 13 weeks and completely dissolved after 35 weeks (245 days), aligning with the 300-day lifespan projected from accelerated degradation tests in DPBS. Histological analysis of organ tissues (heart, lungs, liver, spleen, kidneys, and brain) showed no abnormalities or residual Mo particles, confirming the material’s safety and biocompatibility [91]. These results position Mo-based bioresorbable, wireless, and battery-free electrotherapy systems as a highly efficient platform for enhancing and tracking wound healing in diabetic small animal models.

#### 3.1.2. Natural-Skin-Derived Organohydrogel (SGC@MA-Gel) Triboelectric Nanogenerator-Based Smart Battery-Free and Wireless Bioelectronic Platform

**Figure 5:**
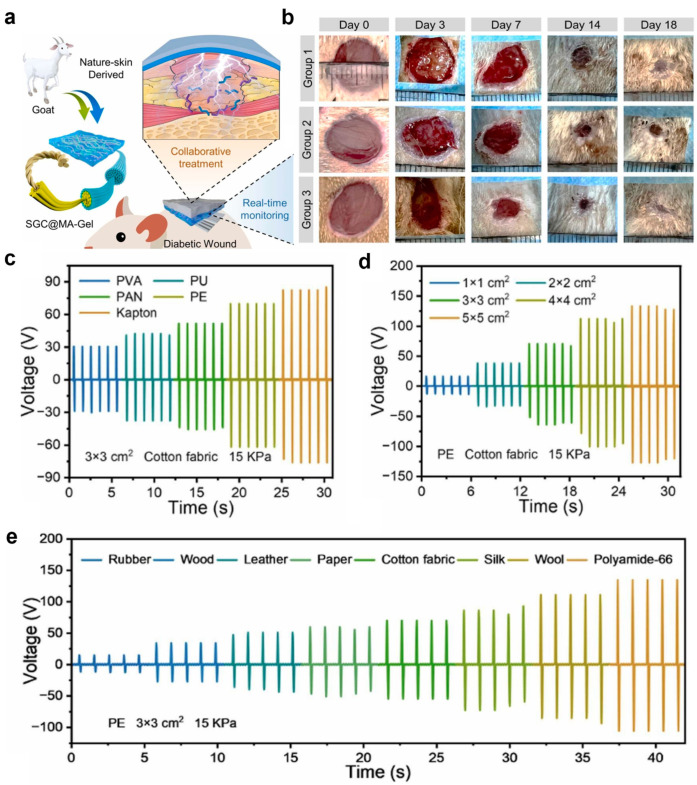
Natural-skin-derived organohydrogel (SGC@MA-Gel) triboelectric nanogenerator-based smart, battery-free, and wireless bioelectronic platform [92]. (**a**) Schematic illustration of the natural-skin-derived organohydrogel (SGC@MA-Gel) triboelectric nanogenerator-based smart, battery-free, and wireless bioelectronic platform. (**b**) In vivo experimental results. (**c**–**e**) Voltage output values under various experimental conditions. Reprinted with permission from Bai et al.; Copyright © 2023 Elsevier Ltd.

The natural-skin-derived organohydrogel (SGC@MA-Gel) triboelectric nanogenerator (S-TENG) is a wireless, battery-free bioelectronic platform designed for integrated wound monitoring, diagnosis, and treatment (Figure 5) [92]. SGC@MA-Gel is synthesized by impregnating pure skin (P-Skin), sourced through traditional leather processing, with a solution containing Q-chitosan, metformin, adenine, HCl, NaCl, Gly, and water in a leather drum [93].

The mechanical characteristics of SGC@MA-Gel, such as tensile strength and elongation at failure, improve with increasing thickness due to a greater density of collagen fibers [94]. With thicknesses ranging from 0.8 mm to 1.6 mm, SGC@MA-Gel aligns with Young’s modulus of human skin (0.5–1.95 MPa), ensuring excellent conformity to wound shapes, comfort, and adaptability to motion. Compared to other wound healing materials, SGC@MA-Gel demonstrates an exceptional tensile strength of 8.14 MPa [95]. The hydrogel exhibits optical transparency (11.9–84.7% in the 400–780 nm visible spectrum), enabling direct wound monitoring and compatibility with biosensors [96]. Gly in the formulation prevents ice formation by blocking hydrogen bonding, maintaining structural integrity at temperatures below −20 °C. Q-chitosan enhances SGC@MA-Gel’s adhesion to polar substrates through hydrogen bonding, coordination bonding, and electrostatic interactions [97]. Tests on porcine skin, muscle, and organ tissues showed an adhesion strength of up to 20 kPa. SGC@MA-Gel maintains superior adhesion on organic and inorganic surfaces, making it highly versatile for multifunctional wound repair and wearable electronic applications. Unlike conventional hydrogel dressings that dry out due to water evaporation, the Gly in SGC@MA-Gel forms strong hydrogen bonds with water molecules, retaining more than 67% of its weight after 7 days. The material also exhibits significant self-healing properties, surpassing those of P-Skin [98].

SGC@MA-Gel naturally resorbs within 2 weeks to 3 months, depending on environmental and body conditions. Its conductivity (0.021 S·m) is facilitated by the presence of Na, H, and Cl ions, making it suitable for electrical applications. S-TENG utilizes SGC@MA-Gel as the electrode material and a negative triboelectric layer for charge transport. When external materials contact the encapsulation layer, charges are induced and flow through the circuit upon separation. Repeated approach-separation cycles generate consistent energy output [99]. Packaged with SGC@MA-Gel and PE film, S-TENG achieves output voltages of 16.1 V, 70.1 V, and 133.0 V, depending on the electrode area (1 × 1 cm^2^, 3 × 3 cm^2^, 5 × 5 cm^2^) (Figure 5c–e) [92]. Stability tests showed minimal voltage degradation after 1000–5000 cycles at frequencies between 0.5 and 8.0 Hz, highlighting its reliability for real-world applications.

Cell viability tests (CCK-8 assay) confirmed excellent cytocompatibility, with over 96.2% viability after 5 days. Q-chitosan also disrupts bacterial cell wall synthesis, providing effective antibacterial properties. No allergic reactions or redness were observed when applied to human skin, confirming its safety and compatibility. SGC@MA-Gel combined with wood, leather, and cotton, among other materials group demonstrated the fastest wound healing, with a wound area reduction of 3% compared to 10% in group 1 and 5% in group 2 after 18 days (Figure 5b) [92]. The results validate the effectiveness of the SGC@MA-Gel S-TENG system as a safe, multifunctional, and biocompatible platform for chronic wound management, with broad applicability in healthcare settings.

#### 3.1.3. Flexible TENG (F-TENG) and Triboelectric-Responsive Drug Delivery Hydrogel (TR-DDH)-Based Wearable Triboelectric Stimulator (WTS)

**Figure 6:**
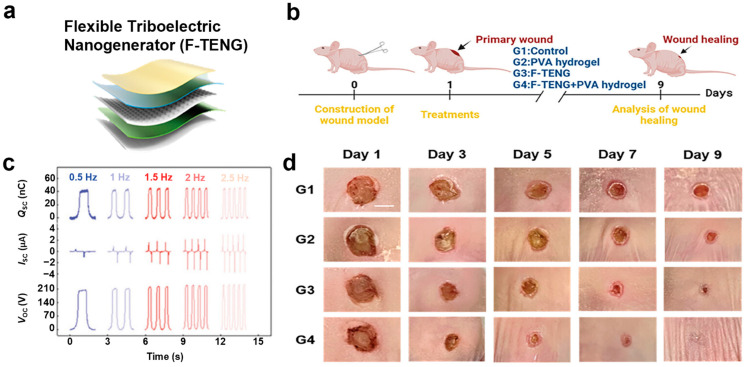
Flexible TENG and triboelectric-responsive drug delivery hydrogel-based wearable triboelectric stimulator [100]. (**a**) Schematic illustration of the F-TENG and TR-DDH-based WTS. (**b**) Voltage, current, and charge output values under various frequency conditions. (**c**) In vivo experimental protocol. (**d**) In vivo experimental results. Reprinted with permission from Qin et al.; Copyright © 2024 Wiley-VCH GmbH.

The wearable triboelectric stimulator (WTS) integrates a flexible triboelectric nanogenerator (F-TENG) with a triboelectric-responsive drug delivery hydrogel (TR-DDH), creating an efficient, synergistic therapeutic platform (Figure 6a) [100]. F-TENG converts mechanical energy, such as human motion, into electrical energy, enabling self-powered electrical stimulation. Meanwhile, TR-DDH facilitates the controlled delivery of curcumin nanoparticles (CURNPs), which enhance antibacterial activity and accelerate wound healing [101]. This system employs PET-ITO films and PVA-PA hydrogel electrodes to generate alternating current through periodic contact-separation movements [102]. Electrical stimulation alters the charge state of PPy, regulating the release of curcumin nanoparticles [103]. The performance of F-TENG was evaluated by measuring open-circuit voltage (V_OC_), short-circuit current (I_SC_), and short-circuit charge (Q_SC_) across a frequency range of 0.5–2.5 Hz. I_SC_ increased from 0.5 to 4 µA as the frequency rose, Q_SC_ remained constant at 40 nC, and V_OC_ reached 240 V at 2.5 Hz (Figure 6b) [100]. The introduction of a conical microstructure in the silicone rubber layer improved the performance of F-TENG [104]. Compared to F-TENG without this structure, it enhanced V_OC_ by 120 V, I_SC_ by 2.2 µA, and Q_SC_ by 24 nC. The microstructure increases the contact area between the two triboelectric layers during friction, thereby boosting charge density and improving overall electrical output.

When attached to the wrist, arm, or knee, the F-TENG produces voltages between 15–20 V as the joints bend. It demonstrates excellent cycling stability, maintaining performance even after more than 20,000 contact-separation cycles. The silicone rubber encapsulating the PVA-PA hydrogel electrode exhibits a high Young’s modulus (1750 kPa) and excellent adhesion to the hydrogel, preventing separation during stretching, bending, or compression [105]. Additionally, the hydrogel electrode shows outstanding electrical conductivity (270 Ω) and significantly slows moisture loss, retaining 96% of its weight after 14 days, showcasing its ability to maintain hydration.

In a full-thickness wound model, the effects of triboelectric stimulation on wound healing were assessed. Mice were divided into four groups: G1 (control), G2 (PVA hydrogel), G3 (F-TENG), and G4 (F-TENG + PVA hydrogel). By day 9, the wounds in G2 had formed scabs, and those in G1 had not fully healed, while the wounds in G3 and G4 healed much more quickly (Figure 6c) [100]. Notably, the wounds in G4 showed the fastest healing, with nearly complete closure by day 9 (wound contraction at 9 days: G1: 50%, G2: 60%, G3: 75%, G4: 95%) (Figure 6d) [100]. The superior tensile strength and adhesion of the PVA-PA hydrogel electrode provided better performance, ensuring stable contact with the skin. The device is designed to resorb gradually over 2–3 weeks after application to the wound, with the resorption rate varying based on the wound environment. Both in vitro and in vivo experiments confirmed that the WTS significantly accelerates the healing of infected wounds, exhibiting strong antibacterial effects and maintaining tissue stability.

### 3.2. Based on Drug Delivery Effects

#### 3.2.1. Panthenol Citrate Biomaterials (PC-PPCN)

**Figure 7:**
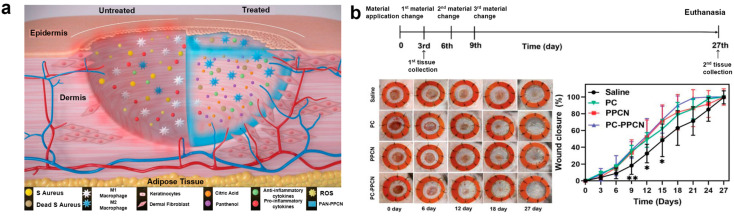
Drug delivery using PC-PPCN [106]. (**a**) Schematic illustration of drug delivery using PC-PPCN (**b**) In vivo experimental results. All data are presented as mean ± SD (*n* = 5; ns, not significant; * *p* < 0.05; ** *p* < 0.01) Reprinted with permission from Wang et al.; Copyright © 2023 The Authors. Advanced Healthcare Materials published by Wiley-VCH GmbH.

Panthenol citrate (PC) is synthesized via a thermal condensation reaction between panthenol and citric acid, targeting wound healing in diabetic patients (Figure 7a) [106,107]. The PC compound is then thermally condensed with citric acid, polyethylene glycol (PEG), and glycerol 1,3-diglycerolate diacrylate (GDD) in a molar ratio of 1.6:1.2:2.1:1, followed by free radical polymerization with N-Isopropylacrylamide (NIPAAM) in a molar ratio of 14.3. This process results in the formation of a thermoresponsive hydrogel, poly(panthenol citrate polyethylene glycol citrate co-N-isopropylacrylamide) (PC-PPCN). The complex ring structure of PC absorbs UV light at 350 nm and emits blue fluorescence at 450 nm, suggesting its potential for bioimaging applications [108]. These fluorescence properties are consistent with previous studies on the fluorescence behavior of chromophores [109].

Characterization of PC-PPCN by differential scanning calorimetry (DSC) and thermogravimetric analysis (TGA) reveals a glass transition temperature (T_g_) of 75.1 °C and high thermal stability, with a decomposition temperature of ~200 °C. As a completely amorphous polymer with a crosslinked structure, PC-PPCN exhibits a low critical solution temperature of 30 °C. This property allows the hydrogel to transition into a conformal gel at body temperature, forming a stable dressing for wound application. Additionally, the hydrogel can be easily removed from the wound with a cold saline solution, reducing the risk of secondary injury [110]. In antioxidant tests, both PC and PC-PPCN demonstrated efficacy comparable to the well-known antioxidant L-ascorbic acid. Within 48 h, they removed over 80% of reactive oxygen species (as measured by the ABTS assay), reduced lipid peroxidation by a factor of five compared to β-carotene, and chelated over 90% of iron ions within 5 min, showing twice the effect of L-ascorbic acid. This superior antioxidant performance is attributed to the aromatic ring structure of PC and PC-PPCN, which stabilizes free radicals more efficiently than conventional antioxidants [111].

Antimicrobial testing using *Staphylococcus aureus* (*S. aureus*), a common wound pathogen, showed that both PC and PC-PPCN reduced bacterial colonies by 98.6% and 93.3%, respectively, confirming their antibacterial activity. MTT analysis demonstrated that even at concentrations up to 10 mg/mL, PC maintained over 80% cell viability, indicating its high biocompatibility. These results suggest that PC and PC-PPCN are biocompatible and suitable for use as safe dressing materials in future in vivo applications. Additionally, both PC and PC-PPCN promoted angiogenesis by enhancing the proliferation, migration, and tube formation of human microvascular endothelial cells (HMVECs). This was evidenced by increased endothelial cell proliferation (compared to saline, *p* < 0.001), reduced wound area (*p* < 0.001), and the formation of junctions, nodules, and networks in a Matrigel environment [112].

To validate these in vitro findings, the therapeutic effects of PC and PC-PPCN were evaluated using a splinted excisional full-thickness wound model in diabetic (db/db) mice. Due to its rapid resorption characteristics in vivo, PC-PPCN required periodic reapplication every 3 days. By day 9, wounds treated with PC and PC-PPCN showed a 65% reduction in wound area, significantly outperforming saline-treated wounds, which showed an 82% reduction. By day 21, the wound treated with PC-PPCN had fully healed, whereas the PC-treated wound was 15% open, and the saline-treated wound remained 30% open (Figure 7b) [106]. In conclusion, both PC and PC-PPCN significantly accelerated the healing of diabetic wounds, with PC-PPCN demonstrating superior efficacy in terms of skin regeneration and wound closure.

#### 3.2.2. Hybrid Biomaterial (Gel@fMLP/SiO_2_-FasL)

**Figure 8:**
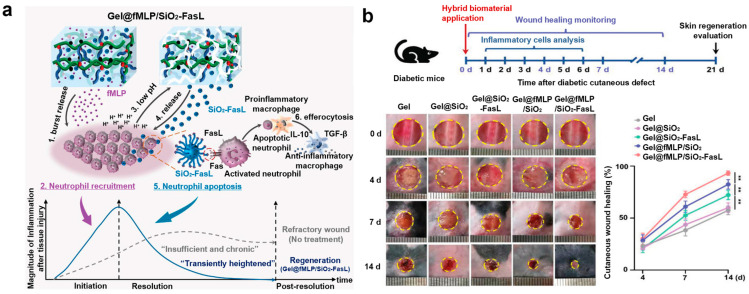
Drug delivery using the hybrid biomaterial (Gel@fMLP/SiO_2_-FasL) [113]. (**a**) Schematic illustration of drug delivery using the hybrid biomaterial (Gel@fMLP/SiO_2_-FasL) (**b**) In vivo experimental results. Statistical analysis was performed by one-way ANOVA. ns, not significant; ** *p* < 0.01. Reprinted with permission from Liu et al.; Copyright © 2022 The Authors. Advanced Science published by Wiley-VCH GmbH.

The phenylboronic acid (PBA)-based polymer hydrogel (Gel@fMLP/SiO_2_-FasL) incorporates formyl-met-leu-phe (fMLP) and FasL-conjugated silica nanoparticles (SiO_2_-FasL), designed to transiently enhance the inflammatory response in chronic, resistant wounds (Figure 8a) [113,114]. This hybrid biomaterial is engineered to precisely control neutrophil recruitment and apoptosis through a two-step process that governs both inflammation initiation and resolution [115]. The first step involves the rapid diffusion of fMLP, a potent neutrophil chemoattractant, into the surrounding environment, where it recruits neutrophils to the wound site. The second step is the pH-sensitive release of FasL-Fas signaling via SiO_2_-FasL, which triggers apoptosis in activated neutrophils within the acidic microenvironment of the hydrogel matrix.

The hydrogel matrix itself features a porous structure with pores ~20 µm in diameter. SEM images reveal numerous SiO_2_ particles embedded within the hydrogel. The hydrogel can load fMLP and SiO_2_-FasL through physical adsorption, and under low pH conditions (e.g., pH 5.5), pH-induced dissociation occurs, ensuring the rapid release of RhB (fMLP) (~75% within 60 min) and SiO_2_-FasL (~80% within 48 h). The hydrogel’s storage modulus (G′) and loss modulus (G″) remain stable across specific deformation ranges, allowing it to withstand substantial deformation while maintaining its intact 3D network structure and high stability [116].

To assess the effectiveness of this hybrid biomaterial in diabetic wounds, various components and hybrid hydrogels were applied once, and wound healing was monitored over 14 days (Gel, Gel@SiO_2_, Gel@SiO_2_-FasL, Gel@fMLP/SiO_2_, Gel@fMLP/SiO_2_-FasL). The wound healing rate was higher in the Gel@SiO_2_-FasL (70%), Gel@fMLP/SiO_2_ (80%), and Gel@fMLP/SiO_2_-FasL (90%) groups compared to the Gel (~55%) and Gel@SiO_2_ (60%) groups, with the Gel@fMLP/SiO_2_-FasL group exhibiting the fastest healing rate (Figure 8b) [113]. Following treatment, the wound length significantly decreased in all groups (~1.45, 1.4, 1.3, 1.25, 1.15 cm), with the Gel@fMLP/SiO_2_-FasL group showing the most effective results. Additionally, Masson’s staining revealed superior collagen deposition and organization in the Gel@fMLP/SiO_2_-FasL group compared to the other groups (~25, 25, 25, 30, 45%). These results indicate that Gel@fMLP/SiO_2_-FasL can effectively enhance the healing of chronic, refractory wounds by modulating a controlled inflammatory response.

#### 3.2.3. Bioactive Hydrogel (OHA-CMC/CNP/EGF)

**Figure 9:**
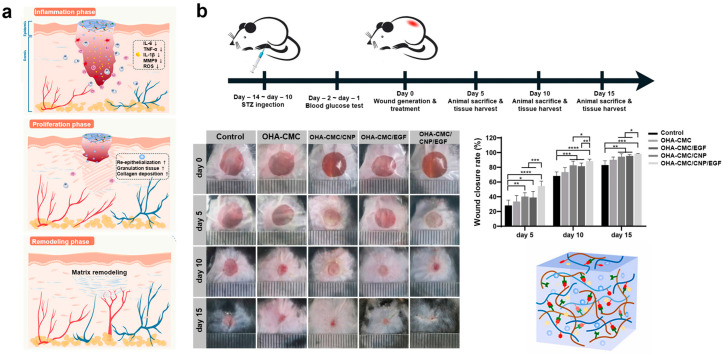
Drug delivery using the bioactive hydrogel (OHA-CMC/CNP/EGF) [117]. (**a**) Schematic illustration of drug delivery using the bioactive hydrogel (OHA-CMC/CNP/EGF) (**b**) In vivo experimental results. The differences were regarded as statistically significant with * *p* < 0.05, ** *p* < 0.01, *** *p* < 0.001, and **** *p* < 0.0001. Reprinted with permission from Hu et al.; Copyright © 2021 The Authors. Publishing services by Elsevier B.V. on behalf of KeAi Communications Co. Ltd., Amsterdam, The Netherlands.

The OHA-CMC hydrogel is synthesized through a reversible Schiff base reaction between aldehyde-modified hyaluronic acid (OHA) and carboxymethyl chitosan (CMC), resulting in a hydrogel with excellent antimicrobial and hemostatic properties (Figure 9a) [117,118]. This hydrogel, when combined with nanotechnologically modified curcumin (CNP) and epidermal growth factor (EGF), releases these bioactive agents sequentially, addressing key challenges in the four stages of diabetic wound healing: hemostasis, inflammation, cell proliferation, and matrix remodeling. Additionally, hydrogel regulates inflammation and oxidative stress while promoting tissue regeneration [119]. Curcumin and EGF exhibit distinct physicochemical properties and dynamic chemical interactions with the hydrogel network. Curcumin is quickly released to mitigate early inflammation and oxidative stress, while EGF is gradually delivered to aid in the later phases of cell proliferation and extracellular matrix (ECM) formation [120].

Rheological analyses of OHA-CMC demonstrate a stable G′ of ~240 Pa, roughly 10 times greater than the G″ in the 0.1–10 Hz range, indicating a robust, crosslinked network structure. Swelling experiments conducted at 37 °C in PBS show that the OHA-CMC hydrogel rapidly swells (equilibrium swelling ratio, ESR: 48–58 g/g), emphasizing its ability to absorb wound exudates and preserve a moist wound environment. SEM images reveal that the hydrogel possesses a highly interconnected porous microstructure with evenly distributed pores, facilitating efficient transport of oxygen, nutrients, and waste exchange [121]. The hydrogel undergoes resorption primarily via surface erosion and bulk resorption, completely resorbing by day 10, demonstrating its bioresorbability and suitability for wound healing applications [122].

Cell viability and proliferation assays using NIH-3T3 fibroblasts show significant increases in cell growth on days 3 and 7 compared to day 1, confirming that the OHA-CMC hydrogel supports cellular proliferation and is biocompatible. CCK-8 assays indicate no cytotoxicity from hydrogel extracts, with cells maintaining 100% viability after 24 h of culture. Furthermore, the hydrogel exhibits a low hemolysis rate of ~0.41%, suggesting good blood compatibility and potential for use in wound dressing applications [123]. The antimicrobial efficacy of the OHA-CMC hydrogel was demonstrated in an *S. aureus* model, where it exhibited a 98.7% reduction in bacterial colony counts compared to the PBS-treated control group. In a mouse hemorrhage model, OHA-CMC rapidly suppressed bleeding, reducing blood loss by 4.7 times compared to the control group (102.1 mg). This hemostatic efficiency surpassed that of commercial hemostatic products, which resulted in only a 3-fold reduction in blood loss [124]. The positively charged amino groups of the hydrogel interact electrostatically with the negatively charged platelets, thereby triggering the coagulation process. In addition to these properties, OHA-CMC/CNP (100 µg) demonstrated potent antioxidant activity, removing 84.6% of 1,1-Diphenyl-2-Picrylhydrazyl (DPPH), indicating its high potential for reducing oxidative stress. Furthermore, OHA-CMC/CNP effectively downregulated cytokine levels, with a low curcumin dose (5 µg) restoring mRNA levels to normal. The hydrogel also accelerated cell migration in vitro, as shown by a scratch assay. OHA-CMC/CNP/EGF reduced the wound area by 28.6% at 12 h and 25.9% at 24 h, promoting faster migration of fibroblasts.

In vivo, the wound healing performance of OHA-CMC/CNP was evaluated in a full-thickness skin wound model in diabetic mice. The OHA-CMC/CNP group exhibited an 88.3% closure rate by day 10, with nearly complete wound closure by day 15 (Figure 9b) [117]. The combination of OHA-CMC/CNP/EGF demonstrated the best therapeutic efficacy, finely regulating drug release in synergy with CNP and EGF, thereby optimizing the wound healing process.

## 4. Conclusions and Future Perspectives

This review provides a comprehensive overview of the bioresorption mechanisms of metal, polymer, and semiconductor materials, highlighting their distinctive characteristics. We also summarized various platforms for wound healing that incorporate electrical stimulation and drug delivery methods using these materials (Table 2). Bioresorbable medical devices offer several advantages, such as eliminating the need for device retrieval after treatment and enabling the use of a broad range of materials—including conductors, semiconductors, and insulators—thereby opening up vast potential for diverse applications [125]. These materials are already being utilized in fields ranging from wound healing to pain management, implantable electrical stimulators, and nerve and bone regeneration [126,127]. However, challenges remain, particularly concerning the long-term stability of bioresorbable materials. Issues such as moisture penetration, immune responses during the resorption process, and the gradual loss of material properties over time require further attention [128,129]. To address these challenges, ongoing research is focused on controlling the resorption rate of bioresorbable materials, enhancing waterproof packaging using oils [130], and developing externally triggered bioresorption techniques [131]. Overall, advancements in bioresorbable materials and devices hold the promise of overcoming the limitations of current implantable and wearable medical devices, potentially leading to the development of innovative new form factors for medical applications.

## Figures and Tables

**Table 1:** Summary of bioresorbable polymers in wound healing applications.

Material	Electrical Conductivity (S m^−^¹)	Weight Loss and Resorption Time	Modulus (MPa)	Elongation (%)	Tensile Strength (MPa)	Ref.
PPy/PDLLA	1.0 × 10^−3^	14% mass loss after 8 weeks	-	-	-	[55,56]
PPy/PLGA or PLLA fibers	1.0 × 10^−16^ to 1.0 × 10^−4^	14–24% mass loss after 12 weeks	-	-	-	[57,58]
PPy/SF	1.1 × 10^2^	82% mass loss after 15 days	-	-	-	[59]
PANI/gelatin	1.4 × 10^−2^ to 2.1 × 10^−2^	50–60% mass loss after 7–14 days	-	-	-	[60]
PEDOT/PLLA	4.7 × 10^−3^	resorption rate of 10% after 8 weeks	-	-	-	[61]
Modified PPy	1 × 10^2^ to 1 × 10^4^	100% (thin film; pH 8.2) mass loss after 24 h 6–27% (pellet; pH 7.2) mass loss after 80 days	-	-	-	[62]
SPT/PEI	2.76 × 10^−2^	100% mass loss after 12–18 weeks	-	-	-	[63]
BCPU	1.2 × 10^−5^ to 5.5 × 10^−5^	12–14% mass loss after 8 weeks	-	-	-	[64]
PGAP	2 × 10^−5^	50% mass loss after 70 days	-	-	-	[65]
PCL	Non-conductive	3% mass loss after 5 weeks	300–400	70–400	10–25	[66]
PVA	Non-conductive	100% mass loss after 30 min	1400–4000	100–250	30–80	[67]
PGA	Non-conductive	Complete mass loss after 3 weeks	7000–12,000	~15–30	600–1000	[68]
PLA	Non-conductive	~64% mass loss after 12 months	2000–3500	~2–10	50–70	[69]
PLGA	Non-conductive	Complete mass loss within 30–50 days	200–10,000	~5–50	20–50	[70]

**Table 2:** Summary of bioresorbable material applications for wound management.

Methods	Voltage	Current	Waveform	Drug Type	Wound Size	Wound Healing Efficiency	Ref.
Electrostimulation	1.1 V	20 µA	DC	-	10 mm	86 ± 10% 15 days	[86]
Electrostimulation	16.1 V	58 µA	AC	-	10 mm	97% 18 days	[92]
Electrostimulation	210 V	3 µA	AC/DC	CURNPs	6 mm	95% 9 days	[100]
Drug delivery	-	-	-	citric acid, panthenol	10 mm	100% 21 days	[106]
Drug delivery	-	-	-	fMLP, SiO_2_-FasL	10 mm	90% 14 days	[113]
Drug delivery	-	-	-	CNP, CGF	8 mm	100% 10 days	[117]

## Data Availability

No new data were created or analyzed in this study.
